# Publisher Correction: Modelling genetic stability in engineered cell populations

**DOI:** 10.1038/s41467-023-39654-4

**Published:** 2023-06-29

**Authors:** Duncan Ingram, Guy-Bart Stan

**Affiliations:** Centre of Excellence in Synthetic Biology and Department of Bioengineering, Imperial College London, London, United Kingdom

**Keywords:** Gene regulatory networks, Synthetic biology, Computational models, Population dynamics

Correction to: *Nature Communications* 10.1038/s41467-023-38850-6, published online 12 June 2023

In the original version of this Article, in the third paragraph of the Discussion the equation ‘$$T=\left(\begin{array}{c}s\\ 2\end{array}\right)\cdot d\cdot {s}^{d-1}$$’ incorrectly presented the equation as ‘$$T=\left(\begin{array}{c}s\\ 2\end{array}\right)\cdot d\cdot {s}^{d1}$$’. In addition, in the fifth paragraph of the Discussion, the term “mutation probability ‘z_M_’ was incorrectly presented as mutation probability ‘z_*M*_’. These have been corrected in both the HTML and PDF versions of the Article.

